# Effectiveness of catch-up and at-birth nirsevimab immunisation against RSV hospital admission in the first year of life: a population-based case–control study, Spain, 2023/24 season

**DOI:** 10.2807/1560-7917.ES.2025.30.5.2400596

**Published:** 2025-02-06

**Authors:** Olivier Núñez, Carmen Olmedo, David Moreno-Perez, Nicola Lorusso, Sergio Fernández Martínez, Pedro Eliseo Pastor Villalba, Ángeles Gutierrez, Marcos Alonso Garcia, Pello Latasa, Rosa Sancho, Jacobo Mendioroz, Montserrat Martinez-Marcos, Enriqueta Muñoz Platón, María Victoria García Rivera, Olaia Pérez-Martinez, Rosa Álvarez-Gil, Eva Rivas Wagner, Nieves López Gonzalez-Coviella, Matilde Zornoza, M Isabel Barranco, M del Carmen Pacheco, Virginia Álvarez Río, Miguel Fiol Jaume, Roxana Morey Arance, Begoña Adiego Sancho, Manuel Mendez Diaz, Noa Batalla, Cristina Andreu, Jesús Castilla, Manuel García Cenoz, Ana Fernández Ibáñez, Marta Huerta Huerta, Ana Carmen Ibáñez Pérez, Belén Berradre Sáenz, Joaquín Lamas, Luisa Hermoso, Susana Casado Cobo, Manuel Galán Cuesta, Sara Montenegro, María Domínguez, Inmaculada Jarrín, Aurora Limia, Roberto Pastor-Barriuso, Susana Monge, the Nirsevimab Effectiveness Study Collaborators, The Nirsevimab Effectiveness Study Collaborators, Inés del Ramo Torreblanca, Ana Lameiras Azevedo, Irene Morales Arjona, Alejandra López Zambrano, M Dolores Lasheras Carbajo, José Francisco Barbas del Buey, Mª Jesús Rodríguez Recio, Ermengol Coma, Luca Basile, María Ángeles Rafael de la Cruz López, Emma Corraliza Infanzón, María-Isolina Santiago-Pérez, María-Teresa Otero-Barrós, Jaime Jesús Pérez Martín, Alonso Sánchez Migallón, Giselle Pérez Suarez, Leticia Bravo Muñoz, Itziar Casado, Guillermo Ezpeleta, Pilar Alonso Vigil, Mario Margolles, Eva Martínez Ochoa, María Merino Díaz, Julián Manuel Domínguez Fernández, Ninoska Lopez Berrios, María Victoria Jiménez Cabanillas, Daniel Castrillejo, Gorka Loroño Ortiz, Koldo López Guridi, Luis Viloria

**Affiliations:** 1 National Centre of Epidemiology, Institute of Health Carlos III, Madrid, Spain; 2 CIBER in Epidemiology and Public Health (CIBERESP), Madrid, Spain; 3 Vaccines Division, General Directorate of Public Health, Ministry of Health, Madrid, Spain; 4 Regional University Hospital – IBIMA, Malaga, Andalusia, Spain; 5 General Directorate of Public Health and Pharmaceutical Regulation, Regional Ministry of Health and Consumption, Seville, Andalusia, Spain; 6 Sub-directorate of Epidemiology and Public Health Surveillance, Valencia, Autonomous Community of Valencia, Spain; 7 General Directorate of Public Health, Madrid, Autonomous Community of Madrid, Spain; 8 Department of Health, Vitoria-Gasteiz, Basque Country, Spain; 9 Department of Health, Public Health Secretariat, Barcelona, Catalonia, Spain; 10 Technical advisor to the General Directorate of Public Health, Toledo, Castilla-La Mancha, Spain; 11 General Directorate of Public Health, Toledo, Castilla-La Mancha, Spain; 12 General Directorate of Public Health, Department of Health, Santiago de Compostela, Galicia, Spain; 13 General Directorate of Public Health, Canary Islands Health Service, Santa Cruz de Tenerife, Canary Islands, Spain; 14 General Directorate of Public Health & Addictions, Murcia, Region of Murcia, Spain; 15 General Directorate of Public Health, Department of Health, Valladolid, Castilla y León, Spain; 16 University Hospital Son Espases, Palma de Mallorca, Balearic Islands, Spain; 17 General Directorate of Public Health, Zaragoza, Aragón, Spain; 18 General Directorate of Public Health, Mérida, Extremadura, Spain; 19 Instituto de Salud Pública de Navarra – IdiSNA, Pamplona, Navarra, Spain; 20 General Directorate of Public & Mental Health, Oviedo, Asturias, Spain; 21 General Directorate of Public Health, Logroño, La Rioja, Spain; 22 University Hospital of Melilla, Melilla, Spain; 23 General Directorate of Public Health, Melilla, Spain; 24 General Directorate of Public Health, Santander, Cantabria, Spain; 25 University Hospital of Ceuta, Ceuta, Spain; 26 CIBER on Infectious Diseases (CIBERINFEC), Madrid, Spain; 27 The individuals are listed under collaborators; * These authors contributed equally to the work and share last authorship.

**Keywords:** respiratory syncytial virus, hospitalisation, Nirsevimab, effectiveness, observational study

## Abstract

**Background:**

Respiratory syncytial virus (RSV) causes substantial morbidity in infants < 1 year. In October 2023, Spain recommended the monoclonal antibody nirsevimab to all children born since 1 April 2023, at birth or as catch-up if born before October 2023.

**Aim:**

We estimated nirsevimab effectiveness in preventing RSV hospitalisations during the 2023/24 season.

**Methods:**

We conducted a nationwide population-based matched case–control study. Cases were children hospitalised for lower respiratory tract infection who were RSV PCR-positive. For each case, we selected four population density controls born in the same province and date (±2 days). We defined at-birth immunisation as receiving nirsevimab during the first 2 weeks of life, and catch-up immunisation within 30 days from campaign onset. Causal intention-to-treat (ITT) and per-protocol (PP) effectiveness was estimated using inverse-probability-of-immunisation weighted conditional logistic regression.

**Results:**

We included 406 cases and 1,623 controls in catch-up and 546 cases and 2,182 controls in at-birth immunisation studies. Effectiveness in preventing RSV hospitalisations for catch-up immunisation was 71% (95% confidence interval (CI): 65–76) by ITT and 80% (95% CI: 75–84) PP. Effectiveness for at-birth immunisation was 78% (95% CI: 73–82) by ITT and 83% (95% CI: 79–87) PP. Effectiveness was similar for ICU admission, need of mechanical ventilation, and RSV viral subgroups A and B. Children born pre-term or with birthweight < 2,500 g showed lower PP effectiveness of 60–70%.

**Conclusions:**

Population-level nirsevimab immunoprophylaxis in children in their first RSV season was very effective in preventing RSV hospitalisations, ICU admission and mechanical ventilation, with reduced but still high effectiveness for pre-term and low-birthweight children.

**Key public health message**

**What did you want to address in this study and why?**

The administration of a long-acting monoclonal antibody, such as nirsevimab, in a population-wide immunisation campaign has no precedent in public health practice. Assessing the effectiveness of this preventive intervention against severe RSV infection in children in their first respiratory virus season is of utmost importance to inform future recommendations.

**What have we learnt from this study?**

The risk of hospitalisation for an RSV infection was reduced by around 80% in children under 6 months who received nirsevimab immunisation. Effectiveness was similar for intensive care admission and need of mechanical ventilation, as well as for different RSV subgroups, while effectiveness was slightly lower for children born pre-term, with low birthweight, or from multiple pregnancy.

**What are the implications of your findings for public health?**

Our results support nirsevimab administration to all children born during or entering their first respiratory virus season and show that the entire newborn population greatly benefited from this intervention, including children with comorbidities or low birthweight and those born pre-term or from multiple pregnancy.

## Introduction

Respiratory syncytial virus (RSV) is among the most common causes of acute lower respiratory tract infection (LRTI) in young children and poses a great burden on healthcare systems during the epidemic season [1]. It is estimated that 26% of healthy term-born infants in Europe experience an RSV infection during their first year of life, 14% seek medical attention, 1.8% are hospitalised and 0.1% require intensive care [2]. Infants younger than 2 months are most at risk of severe disease, with risk decreasing thereafter [3]. In the northern hemisphere, the RSV epidemic season normally extends between October and end of March.

Nirsevimab (Beyfortus) is a monoclonal antibody approved by the European Medicines Agency in October 2022 to prevent serious LRTI caused by RSV in infants born during or entering their first RSV season [4]. It targets the prefusion conformation of the RSV fusion (F) protein at a highly conserved antigen site, neutralising a diverse panel of RSV A and B viral subgroups [5], and has an extended half-life, with geometric mean neutralising antibody concentrations being > 140-fold higher and > 50-fold higher than baseline maternal antibody levels 31 and 151 days after nirsevimab administration, respectively [6]. This allows infants to be protected with a single dose throughout the respiratory virus season.

In autumn 2023, Spain became one of the few countries worldwide, along with Luxembourg, France, the United States and one region in Italy, to recommend systematic administration of nirsevimab to all infants born or entering their first respiratory virus season. For infants born during the RSV season, nirsevimab was indicated as soon as possible after birth, preferably at hospital within the first 48 h of life or otherwise at the first scheduled primary care visit. For children born between 1 April 2023 and campaign onset, catch‐up immunisation was recommended [7]. The recommended dose was 50 mg for those under 5 kg and 100 mg for those weighing ≥ 5 kg at the time of administration. Immunisation was free of charge and coverage reached 92% for at-birth immunisation and 87% for catch-up immunisation [8].

To inform future public health prevention programmes, this study aimed to estimate the effectiveness of nirsevimab in preventing hospital admission due to RSV infection, when indicated at birth or as catch-up immunisation, overall and by newborn characteristics, during the 2023/24 RSV season in Spain.

## Methods

### Study setting, design and eligibility criteria

We implemented a population-based matched case–control study in the 19 autonomous regions of Spain. Cases were children born from 1 April 2023 who were admitted to public hospitals due to LRTI, apnoea or sepsis from the onset of the nirsevimab immunisation campaign in each Spanish region (generally 1 October 2023, for the exact campaign dates by region see Supplementary Table S1) to 31 March 2024, and who tested positive for RSV by PCR 10 days before to 3 days after the admission date. Most regions participated with the whole public hospital network (the main healthcare provider in Spain), while Aragon, Balearic Islands, Catalonia and Extremadura participated only with some public hospitals with better data accessibility, and the Canary Islands included only the three most populated islands. Most autonomous regions recruited all cases fulfilling the case definition, except Andalusia, which selected a convenience sample of all cases until reaching the final sample size. The distribution of cases by region is available in Supplementary Table S2.

Using density (risk-set) sampling, for each case we selected four controls from children born in the same regional hospital network and who had not moved outside the region, died or experienced RSV-associated infection up to the hospitalisation date of the case (matching date). To improve study efficiency and ensure adequate simultaneous control of confounding by province (52 territorial subdivisions of autonomous regions) and date of birth, we additionally matched controls to cases on province and date of birth (± 2 days or exceptionally ± 4 days in small regions of Ceuta and Melilla). Controls were identified from birth registries or from registries of at-birth screening programmes for metabolic diseases, depending on the region. Where time of birth was available, we selected the two children born immediately before and the two born immediately after the case; where it was not available, we selected controls randomly.

Matched sets for cases born before and after the onset of immunisation campaigns in each region were separated into catch-up immunisation and at-birth immunisation case–control studies, respectively. We excluded the region of Navarre from the catch-up study because they did not implement catch-up immunisation. We additionally excluded four regions (Balearic Islands, Basque Country, Extremadura, and Melilla) from the catch-up study because their campaigns began in late October or November, and nirsevimab catch-up administration overlapped substantially with the RSV epidemic in Spain, which started in the second week of November 2023. Campaigns with a late onset will have missed more prevention opportunities and will therefore have a lower estimated effectiveness.

Data were collected from clinical and immunisation records into a study data collection form implemented online using the RedCap software.

### Statistical analysis

We performed a causal case–control analysis. A detailed description and justification of the causal case–control analysis is provided in the Supplementary Methods. Briefly, this causal analysis was based on first emulating a hypothetical target trial in the underlying newborn population through cloning and censoring, and then performing an inverse probability of censoring weighted analysis of nested case–control data from this cloned and censored population [9].

The emulated target trial intervention was the administration of nirsevimab in the first 30 days of campaign for catch-up immunisation or in the first 14 days of life for at-birth immunisation, which matched its real-life implementation; the cumulative proportions of immunised controls over time in the catch-up and at-birth immunisation studies are displayed in Supplementary Figure S1. This intervention grace period produces a lack of alignment between the at-risk time (from campaign onset or from birth) and the intervention time (later during grace period), which results in immortal-time bias (immunised children must remain free from hospitalisation until nirsevimab administration) [10]. To correct this bias, we created two clones per children, assigned each clone to either immunisation or no immunisation, and censored their follow-up when they deviated from the assigned immunisation group. Clones assigned to immunisation were censored at the end of the grace period if they reached that time without receiving nirsevimab, whereas clones assigned to no immunisation were censored at any time they received nirsevimab during the grace period for intention-to-treat (ITT) analysis or during the entire follow-up for per-protocol (PP) analysis; for a graphical representation of the censored follow-up of cloned children we refer to Supplementary Figure S2.

Causal estimates of ITT and PP effectiveness were obtained from conditional logistic regression models relating the assigned immunisation with RSV hospitalisation among cloned cases and controls who remained uncensored at the matching date; the uncensored clones of each case and its selected matched controls are illustrated in Supplementary Figure S3. Since censoring was likely to be informative (immunisation in the original population was not at random; for the main determinants of catch-up and at-birth immunisation see Supplementary Tables S3 and S4), the above models were weighted by the inverse of the probability of clones remaining uncensored up to the matching date [11], given the assigned immunisation, sex (female or male), gestational age (< or ≥ 37 weeks), birth weight (< or ≥ 2,500 g), multiple pregnancy, previous hospitalisation for non-RSV related disease, and previous comorbidities (none or ≥ 1 among those included in nirsevimab recommendation) [7]. To estimate censoring weights, we used a pooled logistic model among controls, treating each child-day as an observation and accounting for matching factors. Weights were stabilised by including in the numerator the probability of remaining uncensored given only baseline factors (at campaign onset or at-birth). Therefore, the weighted conditional logistic models to estimate nirsevimab effectiveness were further adjusted for baseline factors. Conservative 95% confidence intervals (CI) were calculated using robust standard errors [11].

For comparison with other studies, we also obtained pragmatic estimates of effectiveness by naïvely comparing cases and controls according to their actual immunisation status at the matching date and adjusting for risk factors up to that date.

Specific effectiveness in different population groups was estimated by including interaction terms between immunisation and baseline factors, including month of birth, sex, gestational age, birth weight and multiple pregnancy. To assess effectiveness against specific outcomes, we stratified matched sets according to case characteristics, such as co-detection of other virus, intensive care unit (ICU) admission, mechanical ventilation (either invasive or non-invasive) and RSV viral subgroup.

Finally, to assess the overall impact of the nirsevimab immunisation campaign, based on per-protocol estimates of immunisation coverage and effectiveness, we estimated the population prevented fraction as the proportion of all cases of RSV hospitalisation that have been prevented by nirsevimab immunisation [12].

## Results

**Figure 1:**
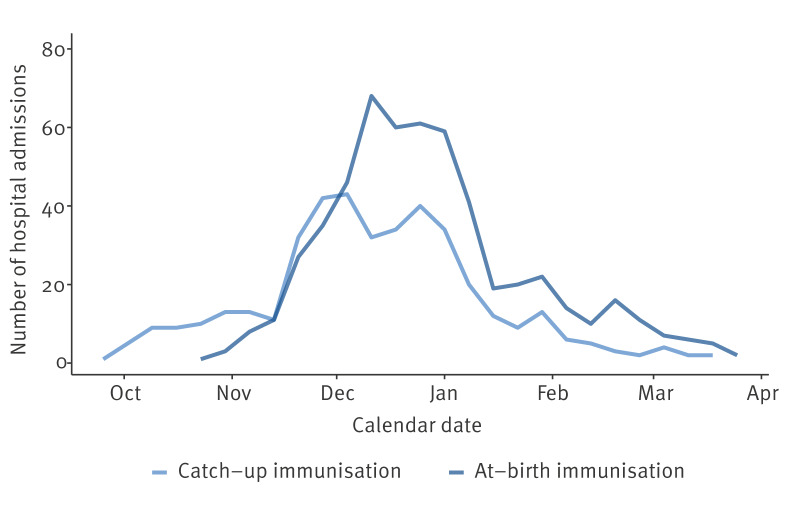
Hospital admissions for respiratory syncytial virus infection, by epidemic week, of children born before (catch-up immunisation) and after (at-birth immunisation) the start of nirsevimab immunisation campaigns, Spain, 2023/24 season (n = 952)

For the catch-up and the at-birth immunisation studies, respectively, we included 406 cases and 1,623 controls and 546 cases and 2,182 controls. Cases distributed in an epidemic wave peaking in December 2023 (Figure 1).

In the catch-up study, 184 cases (45%) and 1,210 controls (75%) received nirsevimab in the first 30 days of the campaign, while 21 cases (5%) and 149 controls (9%) received it later. In the at-birth study, 376 cases (69%) and 1,950 controls (89%) received nirsevimab in the first 2 weeks of life, while 23 cases (4%) and 89 controls (4%) received it later. Median time from nirsevimab receipt to matching was 72 days (interquartile range (IQR): 49–94) and 63 days (IQR: 44–85) in catch-up immunised cases and controls, respectively, and 43 days (IQR: 27–61) and 41 days (IQR: 25–59) in at-birth immunised cases and controls.

Compared with controls, cases were more frequently male, pre-term, with low birthweight, comorbidities and previous non-RSV related hospitalisation, were born from multiple pregnancy, not the mother’s first-born, and with no breastfeeding (Table 1). The RSV viral subgroup A was detected in 36 cases (74% of those with known RSV subgroup) in the catch-up and 38 cases (57% of those with known RSV subgroup) in the at-birth study. There were 69 cases (17%) in the catch-up and 85 cases (16%) in the at-birth study with co-detection of other respiratory virus. Admission to an ICU occurred in 59 cases (15%) in the catch-up immunisation vs 125 cases (23%) in the at-birth study. Mechanical ventilation (either invasive or non-invasive) was required by 115 cases (28%) vs 181 cases (33%). The highest number of cases were children born in October and November 2023, who were ≤ 2 months of age at the epidemic peak; the number of cases decreased with increasing age at the peak (i.e. with earlier month of birth).

**Table 1:** Characteristics of cases and controls in the catch-up and at-birth nirsevimab immunisation studies, Spain, 2023/24 season (n = 4,757)

Characteristic	Catch-up immunisation				At-birth immunisation			
	Cases		Controls		Cases		Controls	
	n	%	n	%	n	%	n	%
Number of participants	406		1,623		546		2,182	
Month of birth								
April 2023	33	8.1	131	8.1	NA			
May 2023	49	12.1	196	12.1				
June 2023	46	11.3	184	11.3				
July 2023	77	19.0	308	19.0				
August 2023	82	20.2	328	20.2				
September 2023	113	27.8	452	27.8	5	0.9	20	0.9
October 2023	6	1.5	24	1.5	202	37.0	806	36.9
November 2023	NA				206	37.7	824	37.8
December 2023					104	19.0	416	19.1
January 2024					23	4.2	92	4.2
February 2024					6	1.1	24	1.1
Median age at hospital admission in days (interquartile range)	140 (97–190)		NA		43 (27–61)		NA	
Sex								
Female	172	42.4	769	47.4	207	37.9	1,046	47.9
Male	234	57.6	854	52.6	339	62.1	1,136	52.1
Gestational age (weeks)								
≥ 37	343	84.9	1,494	92.2	487	89.2	2,020	92.8
< 37	61	15.1	127	7.8	59	10.8	157	7.2
Unknown	2		2		0		5	
Birth weight (g)								
≥ 2,500	349	86.4	1,504	92.7	498	91.2	2,003	92.0
< 2,500	55	13.6	118	7.3	48	8.8	173	8.0
Unknown	2		1		0		6	
Multiple pregnancy								
No	387	95.6	1,597	98.6	526	96.3	2,125	97.8
Yes	18	4.4	22	1.4	20	3.7	47	2.2
Unknown	1		4		0		10	
Only child								
No	203	71.0	638	56.7	292	80.2	854	59.0
Yes	83	29.0	488	43.3	72	19.8	594	41.0
Unknown	120		497		182		734	
Breastfeeding								
Never	60	30.3	140	17.5	65	24.6	254	21.5
Ever	138	69.7	660	82.5	199	75.4	929	78.5
Unknown	208		823		282		999	
Previous non-RSV hospitalisation								
No	322	79.3	1,439	88.7	459	84.1	1,887	86.5
Yes	84	20.7	184	11.3	87	15.9	295	13.5
Previous comorbidities^a^								
None	379	93.3	1,600	98.6	528	96.7	2,167	99.3
≥ 1	27	6.7	23	1.4	18	3.3	15	0.7
Case severity^b^								
Co-detection of other virus	69	17.0	NA		85	15.6	NA	
ICU admission	59	14.5			125	22.9		
Mechanical ventilation	115	28.3			181	33.2		
Deceased	1	0.2			1	0.2		
RSV viral subgroup								
A	36	73.5	NA		38	56.7	NA	
B	13	26.5			29	43.3		
Unknown	357				479			
Nirsevimab administration^c^								
As recommended	184	45.3	1,210	74.6	376	68.9	1,950	89.4
After recommendation	21	5.2	149	9.2	23	4.2	89	4.1
Not administered	201	49.5	264	16.3	147	26.9	143	6.6

ICU: intensive care unit; NA: not applicable; RSV: respiratory syncytial virus.

^a^ Bronchopulmonary dysplasia, congenital heart disease with significant hemodynamic alteration, congenital metabolic disorders, Down syndrome, cystic fibrosis, neuromuscular diseases, pulmonary diseases or respiratory tract malformations that difficult expelling of respiratory secretions, severe immunosuppression due to oncohaematological processes, primary immunodeficiency, HIV confirmed infection or continuous treatment with immunosupressive drugs, and children receiving any type of palliative care.

^b^ Not mutually exclusive categories.

^c^ Nirsevimab administration before the matching date. The recommended period was the first 30 days of campaign for catch-up immunisation and the first 14 days of life for at-birth immunisation.

Matched sets for cases with information on gestational age, birth weight and multiple pregnancy were used for estimations: 403 cases (99.3%) for the catch-up and 546 (100%) for at-birth immunisation. Controls with missing data were also excluded, leaving 1,606 (99.3%) and 2,151 controls (98.6%), respectively. Maternal variables (first-born and breastfeeding) were not considered given the high proportion of missing values. Cloning resulted in 431 (+28) cases and 1,681 (+75) controls in the catch-up and 557 (+11) cases and 2,157 (+6) controls in the at-birth immunisation study.

Effectiveness of nirsevimab by ITT was 71% (95% CI: 65–76) for catch-up and 78% (95% CI: 73–82) for at-birth immunisation (Table 2).

**Table 2:** Effectiveness of catch-up and at-birth nirsevimab immunisation against hospitalisation for respiratory syncytial virus infection in the first year of life, Spain, 2023/24 season (based on data from n = 4,706 children)

	Cases		Controls		% effectiveness (95% CI)
	Immunised	Total	Immunised	Total	
**Catch-up immunisation**					
Pragmatic^a^	204	403	1,344	1,606	87.5 (83.1–90.8)
Intention to treat^b^	213	431	1,277	1,681	71.0 (64.6–76.2)
Per protocol^c^	213	412	1,277	1,539	80.3 (75.3–84.4)
**At-birth immunisation**					
Pragmatic^a^	399	546	2,026	2,151	85.5 (80.5–89.2)
Intention to treat^b^	387	557	1,944	2,157	78.0 (72.7–82.3)
Per protocol^c^	387	534	1,944	2,069	83.1 (78.5–86.8)

CI: confidence intervals.

^a^ Pragmatic estimates of effectiveness (95% CI) were obtained from conditional logistic models based on the actual immunisation status at the matching date and adjusting for sex, gestational age, birth weight, multiple pregnancy, previous non-RSV hospitalisation, and previous comorbidities up to that date. Analyses were based on cases and controls with complete covariate information.

^b^ Causal estimates of intention-to-treat effectiveness (95% CI) were obtained from inverse-probability-of-censoring weighted conditional logistic models based on the assigned immunisation among uncensored clones at the end of the intervention period (the first 30 days of campaign for catch-up immunisation and the first 14 days of life for at-birth immunisation). The increase in the number of cases and controls (28 and 75 for catch-up immunisation and 11 and six for at-birth immunisation, respectively) corresponded to clones who remained uncensored in both arms.

^c^ Causal estimates of per-protocol effectiveness (95% CI) were obtained from inverse-probability-of-censoring weighted conditional logistic models based on the assigned immunisation among uncensored clones up to the matching date. The decrease in the number of cases and controls (19 and 142 for catch-up immunisation and 23 and 88 for at-birth immunisation, respectively) corresponded to clones assigned to no immunisation who received out-of-protocol nirsevimab after the intervention period.

**Figure 2:**
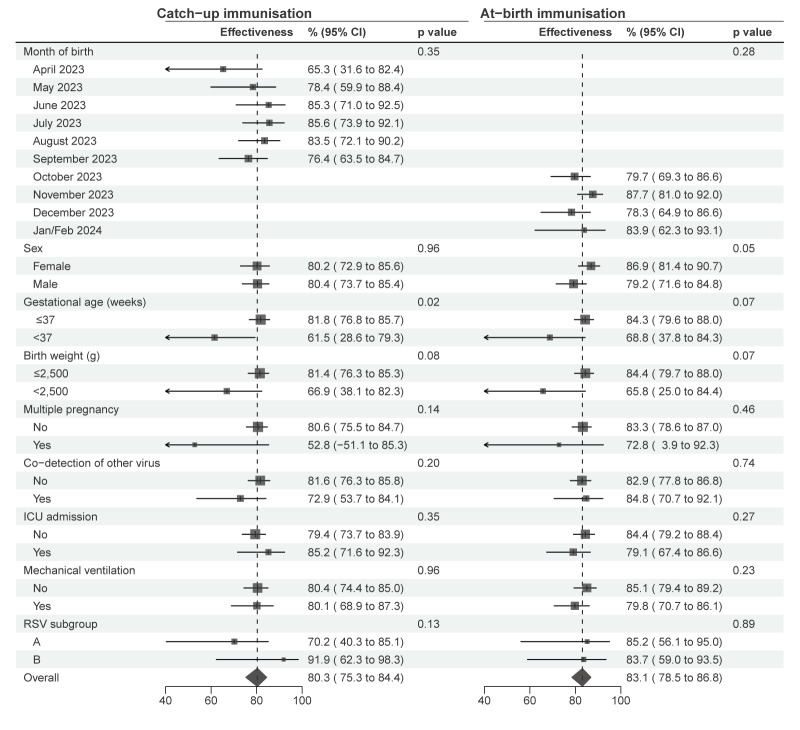
Per-protocol effectiveness of catch-up and at-birth nirsevimab immunisation against hospitalisation for respiratory syncytial virus infection by population groups and case characteristics, Spain, 2023/24 season (n = 4,554 clones)

CI: confidence intervals; ICU: intensive care unit; RSV: respiratory syncytial virus.

Per-protocol estimates were 80% (95% CI: 75–84) for catch-up and 83% (95% CI: 79–87) for at-birth immunisation. The pragmatic effectiveness was slightly higher, at 88% (95% CI: 83–91) for catch-up and 86% (95% CI: 81–89) for at-birth immunisation. Effectiveness PP was high in all analysed sub-groups (Figure 2). Slightly lower effectiveness was observed in male children (only in at-birth immunisation), in pre-term children, or those with low birthweight or born from multiple pregnancy. Effectiveness was similar for more severe outcomes, such as ICU admission and need of mechanical ventilation, as well as for RSV viral subgroups A and B.

Vertical dotted lines represent the point estimate of the overall effectiveness estimation.

Regarding the overall impact of the nirsevimab immunisation campaign, accounting for immunisation coverage and effectiveness, catch-up immunisation prevented 67.8% (95% CI: 62.8–72.2) of hospitalisations for RSV infection among all Spanish children born between 1 April 2023 and the onset of the campaign. Likewise, immunisation with nirsevimab in the first 2 weeks of life prevented 78.1% (95% CI: 73.3–82.0) of hospitalisations for RSV infection among all Spanish children born during the immunisation campaign.

## Discussion

Using a nationwide population-based matched case–control study, analysed with target trial emulation methods, we have estimated that the per-protocol effectiveness of nirsevimab in preventing RSV hospitalisations was 80% (95% CI: 75–84) when administered within the first 30 days of a catch-up immunisation campaign at the season onset and 83% (95% CI: 79–87) when administered in the first 2 weeks of life to children born during the RSV season. Our results also confirm equal effectiveness for RSV viral subgroups A and B and for more severe outcomes, such as ICU admission and need of mechanical ventilation [13].

This observational study also assessed nirsevimab by subgroups, showing generally high effectiveness. Effectiveness seemed lower in pre-term and low-birthweight children, although it was still at around 60–70%. Children born from multiple pregnancy also showed lower, albeit very imprecise, estimates of effectiveness. This could represent a true biological difference in effectiveness if, for example, a higher risk of RSV complications in these children may result in RSV hospitalisation even when immunised. However, it could also reflect some degree of bias, as it is very plausible that medical doctors tend to preferably hospitalise children known to be vulnerable, a hypothesis we could not test in our study. There was also a slightly lower effectiveness in male compared with female children, when immunised at birth. Male children do experience higher RSV hospitalisation and sex differences have been described in the immune response to respiratory viruses and vaccines [14-17]; however, a differential effect of monoclonal antibodies against respiratory viruses has not been documented previously. Moreover, since we did not find a similar sex difference in effectiveness for catch-up immunisation, this finding should be interpreted with caution.

Effectiveness by month of birth was largely similar to the overall estimate. However, the proportion of cases in our study increased as children were born closer to the RSV epidemic peak; thus, we expect the impact of nirsevimab on the absolute number of RSV hospitalisations averted to have been greatest in children born in October and November 2023. Regarding the RSV viral subgroup, there was a difference in point estimates for RSV A and B in the catch-up immunisation study. However, this was based on only 12% of all cases with known viral subgroup, with high imprecision and potential for selection bias, as the reasons for missing subgroup information were not known. Moreover, this difference was not seen in the at-birth immunisation study. Therefore, we consider that there is not sufficient evidence in our results to suggest differences in effectiveness by RSV viral subgroup.

Results are in the range of efficacy observed in clinical trials, from 77% to 83% against RSV hospitalisation and from 76% to 86% against very severe RSV requiring oxygen supplementation [13,18-20]. In our study, intention-to-treat effectiveness was slightly reduced compared with per-protocol effectiveness. This was expected in our target trial emulated with real-world data, since children receiving nirsevimab after the grace period were kept as non-immunised in the ITT analysis. In contrast, a pragmatic estimation merely based on immunisation status at the time of matching, as is most often done in case–control studies, overestimated effectiveness.

Observational studies in different Spanish regions have estimated a crude effectiveness of nirsevimab against RSV hospitalisation of 88% for catch-up immunisation [21] and 70% and 89% for at-birth immunisation [22,23]. A cohort study pooling catch-up and at-birth immunisation estimated 82% effectiveness after adjusting for immunisation group, sex and residential area [24], although time-invariant immunisation status assignment and different calendar time distribution between immunised and non-immunised children could have resulted in certain bias [25,26]. A test-negative case–control study in the United States, where nirsevimab was prioritised for infants younger than 6 months and showed 6% uptake, estimated effectiveness of 90% after adjusting for age at enrolment, month of illness and high-risk medical conditions [27]. In France, a hospital-based case–control study, with 28% nirsevimab uptake among control children who visited the emergency department for non-respiratory diseases, showed 83% effectiveness against hospitalisation for RSV-associated bronchiolitis [28]. Effectiveness against ICU admission has been estimated at between 70% and 90% [21,22,27-29]. While all available estimates are in a compatible range, differences in nirsevimab uptake, population characteristics, relevant confounders adjustment, and study design and analysis may explain variability across studies.

Our study has some limitations. Firstly, it could be affected by residual confounding if common causes of nirsevimab administration and RSV hospitalisation were not accounted for. We collected data ad hoc from hospital records to increase completeness and quality, but the high proportion of missing information on first-born status and breastfeeding prevented accounting for these factors in the models. Moreover, some relevant risk factors related to pregnancy and family socioeconomic conditions could not be collected. Secondly, for feasibility, we used a case–control rather than a cohort design. However, our density (risk-set) sampling of controls allows to obtain unbiased estimates of the hospitalisation rate ratios in the underlying population and the close case–control matching on province and birthdate minimised the potential for selection bias related to the immunisation status [30]. Thirdly, a convenience sample of all available cases was selected in one of the 19 Spanish regions. Nevertheless, we found no systematic differences between cases included and not included in the study among the full set of potential cases from the centralised databases of that region, and hence we assess no substantial impact of the sample selection process on the study validity and representativeness. Finally, we were only able to assess effectiveness of catch-up immunisation in regions that started the campaign at the onset of the RSV season in late September or early October 2023 and in which the campaign hardly overlapped with the epidemic wave. Our results can only be extrapolated to other settings with similar implementation. In regions that started later (Balearic Islands, Basque Country, Extremadura and Melilla), the 30-day grace period defined in our target trial emulation for catch-up immunisation greatly overlapped with the RSV epidemic and, since most cases had occurred before the intervention, the immunisation effectiveness would have been very low by design. When the immunisation campaign starts close to the RSV epidemic, a quicker intervention (i.e. immunising all children eligible for catch-up immunisation within 10–15 days) would need to be defined and implemented to achieve good effectiveness.

## Conclusion

Population-level nirsevimab administration in children born or entering their first RSV season was very effective in preventing RSV hospitalisations, ICU admissions and need of mechanical ventilation. Effectiveness was high, around 80%, for all analysed population groups, although a reduced but still high effectiveness of between 60 and 70% was found for pre-term and low-birthweight children. The effectiveness of nirsevimab, together with its high coverage in Spain, have resulted in a substantial impact of the nirsevimab recommendation, supporting the implementation of similar immunisation campaigns in the upcoming seasons.

## Supplementary Data

678000 Supplementary Material

## References

[1] Shi T McAllister DA O’Brien KL Simoes EAF Madhi SA Gessner BD Global, regional, and national disease burden estimates of acute lower respiratory infections due to respiratory syncytial virus in young children in 2015: a systematic review and modelling study. Lancet. 2017;390(10098):946-58. 10.1016/S0140-6736(17)30938-8 28689664 PMC5592248

[2] Wildenbeest JG Billard MN Zuurbier RP Korsten K Langedijk AC van de Ven PM The burden of respiratory syncytial virus in healthy term-born infants in Europe: a prospective birth cohort study. Lancet Respir Med. 2023;11(4):341-53. 10.1016/S2213-2600(22)00414-3 36372082 PMC9764871

[3] Del Riccio M Spreeuwenberg P Osei-Yeboah R Johannesen CK Fernandez LV Teirlinck AC Burden of respiratory syncytial virus in the European Union: estimation of RSV-associated hospitalizations in children under 5 years. J Infect Dis. 2023;228(11):1528-38. 10.1093/infdis/jiad188 37246724 PMC10681872

[4] Beyfortus | European Medicines Agency [Internet] [Internet]. [cited 2023 Dec 23]. Available from: https://www.ema.europa.eu/en/medicines/human/EPAR/beyfortus

[5] Zhu Q McLellan JS Kallewaard NL Ulbrandt ND Palaszynski S Zhang J A highly potent extended half-life antibody as a potential RSV vaccine surrogate for all infants. Sci Transl Med. 2017;9(388):1928. 10.1126/scitranslmed.aaj1928 28469033

[6] Wilkins D Yuan Y Chang Y Aksyuk AA Núñez BS Wählby-Hamrén U Durability of neutralizing RSV antibodies following nirsevimab administration and elicitation of the natural immune response to RSV infection in infants. Nat Med. 2023;29(5):1172-9. 10.1038/s41591-023-02316-5 37095249 PMC10202809

[7] Ponencia de Programa y Registro de Vacunaciones. Comisión de Salud Pública del Consejo Interterritorial del Sistema Nacional de Salud. Recomendaciones de utilización de nirsevimab frente a virus respiratorio sincitial para la temporada 2023-2024 [Recomendations for the use of nirsevimab against the respiratory syncytial virus for the 2023-2024 season in Spain]. Madrid, July 2023. [Internet]. Available from: https://www.sanidad.gob.es/areas/promocionPrevencion/vacunaciones/comoTrabajamos/docs/Nirsevimab_2023.pdf

[8] Ponencia de Programa y Registro de Vacunaciones. Comisión de Salud Pública del Consejo Interterritorial del Sistema Nacional de Salud. Recomendaciones de utilización de nirsevimab para la temporada 2024-2025 en España [Recomendations for the use of nirsevimab in the 2024-2025 season in Spain]. Madrid, March 2024. Available at: [Internet]. [cited 2024 Jun 21]. Available from: https://www.sanidad.gob.es/areas/promocionPrevencion/vacunaciones/comoTrabajamos/docs/Nirsevimab.pdf

[9] Rose S van der Laan MJ . A targeted maximum likelihood estimator for two-stage designs. Int J Biostat. 2011;7(1):17. 10.2202/1557-4679.1217 21556285 PMC3083136

[10] Hernán MA Sauer BC Hernández-Díaz S Platt R Shrier I . Specifying a target trial prevents immortal time bias and other self-inflicted injuries in observational analyses. J Clin Epidemiol. 2016;79:70-5. 10.1016/j.jclinepi.2016.04.014 27237061 PMC5124536

[11] Robins JM Hernán MA Brumback B . Marginal structural models and causal inference in epidemiology. Epidemiology. 2000;11(5):550-60. 10.1097/00001648-200009000-00011 10955408

[12] Greenland S . Variance estimators for attributable fraction estimates consistent in both large strata and sparse data. Stat Med. 1987;6(6):701-8. 10.1002/sim.4780060607 2825320

[13] Muller WJ Madhi SA Seoane Nuñez B Baca Cots M Bosheva M Dagan R Nirsevimab for prevention of RSV in term and late-preterm infants. N Engl J Med. 2023;388(16):1533-4. 10.1056/NEJMc2214773 37018470

[14] Haerskjold A Kristensen K Kamper-Jørgensen M Nybo Andersen AM Ravn H Graff Stensballe L . Risk factors for hospitalization for respiratory syncytial virus infection: a population-based cohort study of Danish children. Pediatr Infect Dis J. 2016;35(1):61-5. 10.1097/INF.0000000000000924 26398871

[15] Nagayama Y Tsubaki T Nakayama S Sawada K Taguchi K Tateno N Gender analysis in acute bronchiolitis due to respiratory syncytial virus. Pediatr Allergy Immunol. 2006;17(1):29-36. 10.1111/j.1399-3038.2005.00339.x 16426252

[16] Regis E Fontanella S Lin L Howard R Haider S Curtin JA Sex differences in innate anti-viral immune responses to respiratory viruses and in their clinical outcomes in a birth cohort study. Sci Rep. 2021;11(1):23741. 10.1038/s41598-021-03044-x 34887467 PMC8660814

[17] Klein SL Jedlicka A Pekosz A . The Xs and Y of immune responses to viral vaccines. Lancet Infect Dis. 2010;10(5):338-49. 10.1016/S1473-3099(10)70049-9 20417416 PMC6467501

[18] Simões EAF Madhi SA Muller WJ Atanasova V Bosheva M Cabañas F Efficacy of nirsevimab against respiratory syncytial virus lower respiratory tract infections in preterm and term infants, and pharmacokinetic extrapolation to infants with congenital heart disease and chronic lung disease: a pooled analysis of randomised controlled trials. Lancet Child Adolesc Health. 2023;7(3):180-9. 10.1016/S2352-4642(22)00321-2 36634694 PMC9940918

[19] Griffin MP Yuan Y Takas T Domachowske JB Madhi SA Manzoni P Single-dose nirsevimab for prevention of RSV in preterm infants. N Engl J Med. 2020;383(5):415-25. 10.1056/NEJMoa1913556 32726528

[20] Drysdale SB Cathie K Flamein F Knuf M Collins AM Hill HC Nirsevimab for prevention of hospitalizations due to RSV in infants. N Engl J Med. 2023;389(26):2425-35. 10.1056/NEJMoa2309189 38157500

[21] Coma E Martinez-Marcos M Hermosilla E Mendioroz J Reñé A Fina F Effectiveness of nirsevimab immunoprophylaxis against respiratory syncytial virus-related outcomes in hospital and primary care settings: a retrospective cohort study in infants in Catalonia (Spain). Arch Dis Child. 2024;109(9):736-41. 10.1136/archdischild-2024-327153 38857952 PMC11347209

[22] López-Lacort M Muñoz-Quiles C Mira-Iglesias A López-Labrador FX Mengual-Chuliá B Fernández-García C Early estimates of nirsevimab immunoprophylaxis effectiveness against hospital admission for respiratory syncytial virus lower respiratory tract infections in infants, Spain, October 2023 to January 2024. Euro Surveill. 2024;29(6):2400046. 10.2807/1560-7917.ES.2024.29.6.2400046 38333937 PMC10853977

[23] Ezpeleta G Navascués A Viguria N Herranz-Aguirre M Juan Belloc SE Gimeno Ballester J Effectiveness of nirsevimab immunoprophylaxis administered at birth to prevent infant hospitalisation for respiratory syncytial virus infection: a population-based cohort study. Vaccines (Basel). 2024;12(4):383. 10.3390/vaccines12040383 38675765 PMC11054679

[24] Ares-Gómez S Mallah N Santiago-Pérez MI Pardo-Seco J Pérez-Martínez O Otero-Barrós MT Effectiveness and impact of universal prophylaxis with nirsevimab in infants against hospitalisation for respiratory syncytial virus in Galicia, Spain: initial results of a population-based longitudinal study. Lancet Infect Dis. 2024;24(8):817-28. 10.1016/S1473-3099(24)00215-9 38701823

[25] Lund LC Støvring H Pottegård A Andersen M Hallas J . Cox regression using a calendar time scale was unbiased in simulations of COVID-19 vaccine effectiveness & safety. J Clin Epidemiol. 2023;156:127-36. 10.1016/j.jclinepi.2023.02.012 36806733 PMC9933854

[26] Robins J, Hernan M. Estimation of the causal effects of time-varying exposure. In: Longitudinal Data Analysis.2008. p. 553-99.

[27] Moline HL Tannis A Toepfer AP Williams JV Boom JA Englund JA Early estimate of nirsevimab effectiveness for prevention of respiratory syncytial virus-associated hospitalization among infants entering their first respiratory syncytial virus season - New Vaccine Surveillance Network, October 2023-February 2024. MMWR Morb Mortal Wkly Rep. 2024;73(9):209-14. 10.15585/mmwr.mm7309a4 38457312 PMC10932582

[28] Assad Z Romain AS Aupiais C Shum M Schrimpf C Lorrot M Nirsevimab and hospitalization for RSV bronchiolitis. N Engl J Med. 2024;391(2):144-54. 10.1056/NEJMoa2314885 38986058

[29] Paireau J Durand C Raimbault S Cazaubon J Mortamet G Viriot D Nirsevimab effectiveness against cases of respiratory syncytial virus bronchiolitis hospitalised in paediatric intensive care units in France, September 2023-January 2024. Influenza Other Respir Viruses. 2024;18(6):e13311. 10.1111/irv.13311 38840301 PMC11154801

[30] Li G Gerlovin H Figueroa Muñiz MJ Wise JK Madenci AL Robins JM Comparison of the test-negative design and cohort design with explicit target trial emulation for evaluating COVID-19 vaccine effectiveness. Epidemiology. 2024;35(2):137-49. 10.1097/EDE.0000000000001709 38109485 PMC11022682

