# Erratum for Euro Surveill. 2025;30(5)

**DOI:** 10.2807/1560-7917.ES.2025.30.13.250403e

**Published:** 2025-04-03

**Authors:** Eurosurveillance editorial team

**Affiliations:** 1 European Centre for Disease Prevention and Control (ECDC), Stockholm, Sweden

Figure 2 in this article ‘Effectiveness of catch-up and at-birth nirsevimab immunisation against RSV hospital admission in the first year of life: a population-based case–control study, Spain, 2023/24 season’ contained two typographical mistakes at original publication on 6 February 2025: Gestational age (weeks) ≤ 37 and Birth weight (g) ≤ 2,500 have now been corrected to read, respectively, ≥ 37 and ≥ 2,500. These corrections were made on 28 March 2025. We apologise for the mistake.

