# Supplementary material for: Effectiveness of catch-up and at-birth nirsevimab immunisation against RSV hospital admission in the first year of life: a population-based case–control study, Spain, 2023/24 season
Source: Euro Surveill. 2025 Feb 6;30(5):2400596. doi: 10.2807/1560-7917.ES.2025.30.5.2400596 (PMC11803741; doi:10.2807/1560-7917.ES.2025.30.5.2400596)
Supplement: Supplementary Material [file 24-00596_MONGE_Supplement.pdf]

SUPPLEMENTARY MATERIAL

Supplement to: Núñez O, Olmedo C, Moreno-Perez D, Lorusso N, Fernández Martínez S, Pastor Villalba PE, et al. Effectiveness of catch-up and at-birth nirsevimab immunisation against RSV hospital admission in the first year of life: a population-based case-control study, Spain, 2023/24 season. Euro Surveill. 2025.

This supplementary material is hosted by *Eurosurveillance* as supporting information alongside the above mentioned article, on behalf of the authors, who remain responsible for the accuracy and appropriateness of the content. The same standards for ethics, copyright, attributions and permissions as for the article apply. Supplements are not edited by *Eurosurveillance* and the journal is not responsible for the maintenance of any links or email addresses provided therein.

Table of contents

Supplementary Methods 2
Supplementary Results 10
Supplementary References 12
Supplementary Table S1 13
Supplementary Table S2 14
Supplementary Table S3 15
Supplementary Table S4 16
Supplementary Figure S1 17
Supplementary Figure S2 18
Supplementary Figure S3 19
Supplementary Figure S4 20

Supplementary Methods

In this supplement, we describe the steps followed for the causal analysis of the effectiveness of nirsevimab immunisation against hospitalisation for respiratory syncytial virus (RSV) infection in a nationwide population-based matched case-control study in Spain. We first emulated a hypothetical target trial in the underlying population through cloning and censoring, and then performed an inverse probability of censoring weighted analysis of matched case-control data from this cloned and censored population.

This supplement mainly covers the methods for the per-protocol causal analysis of the effectiveness of nirsevimab immunisation at birth. The intention-to-treat analysis followed similar lines, except that it did not account for any nirsevimab administration beyond the initial target immunisation period. Per-protocol and intention-to-treat analyses of the effectiveness of catch-up immunisation were largely similar, except that follow-up of the emulated target trial started on the first day of nirsevimab immunisation campaigns and the target immunisation period was longer. The modifications introduced for these analyses are outlined at the end of the Supplementary Methods.

Target trial emulation in the underlying population

We can use observational follow-up data from the underlying population to emulate an unbiased per-protocol analysis of a hypothetical target trial on the effectiveness of nirsevimab immunisation against RSV hospitalisation. The treatment strategies being compared were immunisation with nirsevimab in the first two weeks of life (target immunisation period) versus no immunisation.

Assuming no losses to follow-up until the end of the RSV season, newborns may experience eight different combinations of immunisation (within the recommended period of 0–13 days of life, ≥14 days of life, or no immunisation up to the season end) and subsequent hospitalisation during follow-up (0–13 days, ≥14 days, or no hospitalisation up to the season

end), as displayed in Supplementary Figure S2A. If we naively compared the rate of hospitalisation for RSV infection between newborns who actually received nirsevimab immunisation (types 1–5 in Supplementary Figure S2A) and those who did not (types 6–8), we would likely overestimate immunisation effectiveness because newborns receiving immunisation necessarily remained free from hospitalisation until nirsevimab administration. To address this immortal-time bias,[1] we created two clones per newborn, assigned each clone to either immunisation or no immunisation with nirsevimab during the first two weeks of life, and artificially censored their follow-up when they deviated from the assigned immunisation (Supplementary Figure S2B). Thus, clones 4*a*, 5*a*, 7*a*, and 8*a* assigned to immunisation were censored at two weeks without receiving nirsevimab (clone 6*a* was not censored because it was hospitalised prior to completing the two-week immunisation period), whereas clones 1*b*, 2*b*, and 3*b* assigned to no immunisation were censored at the time of receiving nirsevimab during the first two weeks. In addition, clones 4*b* and 5*b* were censored due to out-of-protocol immunisation after the first two weeks.

A per-protocol analysis of censored follow-up in the cloned population would be free from immortal-time bias, but prone to selection bias due to informative censoring.[2] If there were risk factors for severe RSV infection that predisposed newborns to receive nirsevimab in the underlying population, uncensored clones who received nirsevimab in the immunisation arm would be at higher baseline risk of hospitalisation than censored clones who did not. Similarly, uncensored clones who did not receive nirsevimab in the no immunisation arm would have lower baseline risk of hospitalisation than censored clones who received it. As a result, the hospitalisation rate ratio comparing uncensored clones assigned to immunisation and no immunisation would underestimate the true immunisation effectiveness.

To correct this selection bias, we can assign weights to uncensored clones equal to the inverse of their probability of remaining uncensored during follow-up, conditional on the

assigned immunisation and previous risk factors for both hospitalisation and censoring.[3] To this end, we determined the probability that newborn j in population stratum i not previously hospitalised for RSV infection remained unimmunised with nirsevimab at the end of day $k \geq 0$, first conditional on its baseline risk factors at birth,

$$\pi^0_{ijk} = P(X_{ijk} = 0|\mathbf{I}_i, \mathbf{Z}_{ij}, D_{ijk\text{-}1} = 0)$$

$$= \prod_{l=0}^{k} P(X_{ijl} = 0|X_{ijl\text{-}1} = 0, \mathbf{I}_i, \mathbf{Z}_{ij}, D_{ijl\text{-}1} = 0),$$

and then conditional on its full risk factor history up to the previous day $k - 1$,

$$\pi^1_{ijk} = P(X_{ijk} = 0|\mathbf{I}_i, \mathbf{Z}_{ij}, \bar{\mathbf{V}}_{ijk\text{-}1}, D_{ijk\text{-}1} = 0)$$

$$= \prod_{l=0}^{k} P(X_{ijl} = 0|X_{ijl\text{-}1} = 0, \mathbf{I}_i, \mathbf{Z}_{ij}, \bar{\mathbf{V}}_{ijl\text{-}1}, D_{ijl\text{-}1} = 0),$$

where X_{ijk} and D_{ijk} were indicators of having received nirsevimab and having been hospitalised for RSV infection by the end of day k, respectively, and $\bar{\mathbf{V}}_{ijk} = (\mathbf{V}_{ij0}, \mathbf{V}_{ij1},\ldots, \mathbf{V}_{ijk})$ was the history of time-varying risk factors up to day k, including indicators of having been previously hospitalised for non-RSV related disease and having been previously diagnosed with any high-risk comorbidity, with $X_{ij,\text{-}1} = D_{ij,\text{-}1} = 0$ and $\bar{\mathbf{V}}_{ij,\text{-}1} = \mathbf{0}$ before birth. Time-constant risk factors $\mathbf{Z}_{ij}$ included sex (female or male), gestational age (< or ≥37 weeks), birthweight (< or ≥2500 g), and multiple pregnancy (no or yes), and $\mathbf{I}_i$ were indicators for each population stratum formed by cross-classifying province and birthdate.

Cloned newborns allocated to immunisation were censored at two weeks of life if they reached the end of this target immunisation period without receiving nirsevimab (top of Supplementary Figure S2B). Censoring due to protocol deviation in this arm was not possible at any other time. Thus, in the immunisation arm, uncensored clone $c = a$ of newborn j in stratum i was assigned the inverse probability of censoring weight $W_{ijck} = 1$ at day $0 \leq k \leq 13$ and $W_{ijck} = (1 - \pi^0_{ij13})/(1 - \pi^1_{ij13})$ at day $k \geq 14$. On the other hand, cloned newborns allocated to no immunisation were censored at any time they received nirsevimab (bottom of

Supplementary Figure S2B). Therefore, in the no immunisation arm, uncensored clone $c = b$ of newborn j in stratum i was assigned the inverse probability of censoring weight $W_{ijck} = \pi^0_{ijk}/\pi^1_{ijk}$ at day $k \geq 0$. Inverse probability of censoring weights assigned to uncensored clones then varied by immunisation arm and follow-up day as

$$W_{ijck} = \begin{cases} 1, & c = a, 0 \leq k \leq 13, \\ (1 - \pi^0_{ij13})/(1 - \pi^1_{ij13}), & c = a, k \geq 14, \\ \pi^0_{ijk}/\pi^1_{ijk}, & c = b, k \geq 0. \end{cases}$$

The denominator of these weights corresponded to the probability of clones remaining uncensored (being immunised at two weeks in the immunisation arm and remaining unimmunised over time in the no immunisation arm) conditional on their full risk factor history. The numerator, which represented the probability of remaining uncensored conditional only on baseline risk factors, was added to stabilise weights and reduce variance in the weighted estimate of immunisation effectiveness. The censoring weights W_{ijck} created a pseudo-population of clones in which, given population stratum and baseline risk factors $\mathbf{Z}_{ij}$, the censoring at day k was not affected by prior follow-up risk factors $\overline{\mathbf{V}}_{ijk\text{-}1}$, thus correcting the selection bias induced by informative censoring with respect to these time-varying factors.[3]

Assuming that all relevant determinants of both nirsevimab immunisation and severe RSV infection were measured and included in $\mathbf{Z}_{ij}$ and $\mathbf{V}_{ijk}$, an unbiased estimate of immunisation effectiveness can be obtained from a weighted stratified Cox proportional hazards model for the rate of hospitalisation for RSV infection given the assigned immunisation and baseline risk factors among cloned and censored newborns with time-varying weights W_{ijck}.[3,4] Note that the selection bias due to time-varying determinants of censoring was controlled by weighting, whereas the selection bias due to baseline determinants was controlled through standard adjustment.

Causal analysis of case-control data

As detailed above, causal inference on the effectiveness of nirsevimab immunisation could be derived from an inverse probability of censoring weighted analysis of a cloned and censored observational cohort from the underlying population.[1] However, as full cohort data were not available, we instead performed a weighted analysis of nested case-control data from this cloned and censored cohort.[5]

From the target population of newborns, we originally selected incident cases of hospitalisation for RSV infection and a sample of four non-hospitalised controls matched to each case on calendar date (density sampling), birthdate, and province, which corresponded to case-control matching on at-risk time, birthdate, and province when using age as the analysis time scale (Supplementary Figure S3A). Clones of each case and its selected matched controls who remained uncensored at the time the case was hospitalised (that is, clones assigned to immunisation who were actually –or could still be– immunised at two weeks and clones assigned to no immunisation who remained unimmunised up to that time) then constituted a sample of the full risk set at that time in a cloned and censored cohort stratified by province and birthdate (Supplementary Figure S3B). Note that some sampled risk sets of uncensored clones might end up with no cases (such as risk sets for cases immunised after the first two weeks of life –newborn 4 in Supplementary Figure S2A–, in which both cloned cases were censored –clones 4*a* and 4*b* in Supplementary Figure S2B–) and thus contribute nothing to the analysis. More importantly, due to the informative censoring of clones, this procedure likely resulted in a selective sample of risk sets at any given time compared with a hypothetical target trial with stratified random immunisation assignment, full adherence, and no loss to follow-up.

In this nested case-control study, the probability that a cloned newborn within each province-by-birthdate stratum were included in the risk set at a given time was equal to the

product of the probability of newborn being selected from the original at-risk population in that stratum and the probability of its clone not being censored by that time. Since all at-risk newborns within each stratum of the original population had equal chance of being selected as controls, the conditional probability of clones being included in a risk set at day k, given the same stratum and baseline risk factors, was inversely proportional to the censoring weights W_{ijck}. Thus, an unbiased estimate of immunisation effectiveness can be obtained by fitting a weighted conditional logistic model to uncensored clones in the sampled risk sets $\mathscr{R}_{it}$ at each event time t in stratum i,

$$\text{logit}\{P(D_{ijct} = 1|\mathscr{R}_{it}, A_{ijc}, \mathbf{Z}_{ij})\} = \beta_{0it} + \beta_1 A_{ijc} + \boldsymbol{\beta}_2'\mathbf{Z}_{ij},$$

with censoring weights W_{ijck} for clones in risk sets at follow-up day k. The risk set-specific intercepts β_{0it} were nuisance parameters determined by the proportion of hospitalised clones $D_{ijct} = 1$ in each risk set, β_1 was a constant coefficient associated with the immunisation assigned to clones $A_{ijc} = 1$ if $c = a$ and 0 if $c = b$, and $\boldsymbol{\beta}_2$ were constant coefficients for baseline risk factors $\mathbf{Z}_{ij}$. In particular, $\exp(\hat{\beta}_1)$ provided an estimate of the causal rate ratio of hospitalisation for RSV infection comparing newborns immunised with nirsevimab in the first two weeks of life with those not immunised during the RSV season. Potential heterogeneity in the hospitalisation rate ratios associated to immunisation by month of birth and baseline risk factors was evaluated by including product terms of immunisation assignment A_{ijc} with level indicators of these factors in the above weighted conditional logistic model. Conservative confidence intervals (CIs) for hospitalisation rate ratios were calculated using robust variance estimates of model coefficients[6] to account for the correlation induced by weighting, the slight artificial increase in the number of cases due to cloning (clones of cases hospitalised during the first two weeks without having been immunised remained uncensored in both arms – clones 6*a* and 6*b* in Supplementary Figure S2B–), and the uncertainty in weight estimation (see below).

To obtain the inverse probability of censoring weights W_{ijck}, we need to estimate consistently the conditional probabilities π^0_{ijk} and π^1_{ijk} of newborns in each population stratum remaining unimmunised with nirsevimab over their time at risk. These probabilities are usually estimated using full cohort data,[3,4] but they can also be estimated using our density (risk-set) sample of controls within each province-by-birthdate stratum, since these controls were representative of the immunisation experience over time within strata of the newborn population.[7] Specifically, the factors in π^1_{ijk} were estimated from a stratified pooled logistic model for the probability that control j in population stratum i remained unimmunised with nirsevimab at the end of any single day $l \geq 0$ up to its selection among those controls unimmunised at previous days,

$$\text{logit}\{P(X_{ijl} = 0 | X_{ijl\text{-}1} = 0, \mathbf{I}_i, \mathbf{Z}_{ij}, \overline{\mathbf{V}}_{ijl\text{-}1}, D_{ijl\text{-}1} = 0)\}$$
$$= \alpha_0 + a_{0p} + \boldsymbol{\alpha}_1'\mathbf{s}(B_{ij}) + \boldsymbol{\alpha}_2'\mathbf{I}(l) + \boldsymbol{\alpha}_3'\mathbf{Z}_{ij} + \alpha_4 V_{1ijl\text{-}1} + \boldsymbol{\alpha}_5'\mathbf{I}(l) V_{2ijl\text{-}1}.$$

Note that, to obtain consistent estimates of the immunisation probabilities, we did not specify separate intercepts for each sparse province-by-birthdate stratum i, but rather used random intercepts a_{0p} for each province p and natural cubic splines of birthdate $\mathbf{s}(B_{ij})$ with three internal knots at the 27.5th, 50th, and 72.5th percentiles and boundary knots at the 5th and 95th percentiles.[4] We also allowed for specific indicators $\mathbf{I}(l)$ of follow-up intervals 0, 1, 2, 3–6, 7–13, and ≥14 days. In this model, $\boldsymbol{\alpha}_3$ were the coefficients associated with baseline risk factors $\mathbf{Z}_{ij}$, α_4 was the coefficient for the presence of comorbidities at previous day $V_{1ijl\text{-}1}$, and $\boldsymbol{\alpha}_5$ were the coefficients for interaction terms between non-RSV hospitalisation at previous day $V_{2ijl\text{-}1}$ and indicators $\mathbf{I}(l)$ of aggregated follow-up intervals 0–2, 3–6, and ≥7 days. Note that immunisation with nirsevimab at day l depended on the history of time-varying risk factors through their values at previous day, and that the effect of previous non-RSV hospitalisation varied with follow-up interval. The factors in π^0_{ijk} were estimated from the

same stratified pooled logistic model removing terms $V_{1ijl\text{-}1}$ and $\mathbf{I}(l)V_{2ijl\text{-}1}$ associated with time-varying factors.

Intention-to-treat analysis

The intention-to-treat analysis of the effectiveness of nirsevimab immunisation at birth was similar to the above per-protocol analysis, except that clones assigned to no immunisation who received nirsevimab after the first two weeks of life (clones 4*b* and 5*b* in Supplementary Figure S2B) should remain uncensored. Thus, matched risk sets for cases hospitalised at follow-up day *k* contributed to the intention-to-treat analysis with their clones assigned to immunisation who received nirsevimab (or could still have received it) during the first two weeks of life and their clones assigned to no immunisation who did not receive nirsevimab during this initial two-week period, with inverse probability of censoring weights

$$W_{ijck} = \begin{cases} 1, & c = a,\ 0 \le k \le 13, \\ (1 - \pi^0_{ij13})/(1 - \pi^1_{ij13}), & c = a,\ k \ge 14, \\ \pi^0_{ijk}/\pi^1_{ijk}, & c = b,\ 0 \le k \le 13, \\ \pi^0_{ij13}/\pi^1_{ij13}, & c = b,\ k \ge 14. \end{cases}$$

Catch-up immunisation effectiveness

Per-protocol and intention-to-treat analyses of the effectiveness of catch-up immunisation among children born before the onset of nirsevimab immunisation campaigns were largely similar. The main differences were that follow-up of the emulated target trial began on the first day of the immunisation campaign and that the treatment strategies being compared were immunisation with nirsevimab in the first 30 days of campaign versus no immunisation. In addition, due to the different target immunisation period, the pooled logistic model for the probability of children remaining unimmunised over time included indicators $\mathbf{I}(l)$ of follow-up intervals 0–6, 7–14, 15–29, 30–59, 60–89, and ≥90 days for the main effect and aggregated

follow-up intervals 0–29 and ≥30 days for their interaction with previous non-RSV hospitalisation $V_{2ijl\text{-}1}$.

Supplementary Results

Factors associated with at-birth and catch-up immunisation

Supplementary Tables S3 and S4 show the odds ratios for remaining unimmunised over time across categories of baseline and time-varying factors, as estimated from the above pooled logistic models among previously unimmunised controls. For nirsevimab immunisation at birth (first columns of Supplementary Table S4), the odds of remaining unimmunised increased by 50% (95% CI, 12% to 99%) in pre-term newborns (<37 weeks of gestation) and by 83% (95% CI, 37% to 144%) in those with low birthweight (<2500 g), with no differences in immunisation by sex and multiple pregnancy. The odds of non-immunisation was also 5.41 times higher (95% CI, 1.89 to 15.5) in infants with previous comorbidities. Newborns with prior hospitalisation for non-RSV related disease postponed nirsevimab immunisation compared with healthy newborns, with odds ratios for non-immunisation of 3.05 (95% CI, 2.16 to 4.31) during days 0–2 of life, 0.85 (95% CI, 0.59 to 1.22) during days 3–6, and 0.28 (95% CI, 0.19 to 0.42) beyond the first week of life.

We observed weaker associations for catch-up immunisation among children born before the onset of the immunisation campaign (first columns of Supplementary Table S3). The odds of remaining unimmunised was 33% lower (95% CI, 60% lower to 12% higher) in children born from multiple pregnancy, with no relevant immunisation differences by sex, gestational age, birthweight, and previous comorbidities. Immunisation during the first 30 days of campaign was similar in children with and without previous non-RSV hospitalisation, but the odds of non-immunisation in previously hospitalised children tended to be 1.57 times higher (95% CI, 0.76 to 3.26) thereafter.

Inverse probability of censoring weights

In the at-birth immunisation study, the mean (range) inverse probability of censoring weights were 1.00 (0.47–3.25) among cases and 1.00 (0.15–3.83) among controls for intention-to-treat analysis, and 1.00 (0.52–5.28) among cases and 1.00 (0.14–12.66) among controls for per-protocol analysis (Supplementary Figure S4). These weights provided an effective control for time-varying risk factors since, given population stratum and baseline risk factors, immunisation with nirsevimab was unrelated with previous non-RSV hospitalisation and comorbidities in the weighted sample of controls (last columns of Supplementary Table S4).

In the catch-up immunisation study, inverse probability of censoring weights were less dispersed, with mean (range) of 1.00 (0.83–1.20) among cases and 1.00 (0.91–1.33) among controls for intention-to-treat analysis, and 1.00 (0.82–1.20) among cases and 1.00 (0.85–1.26) among controls for per-protocol analysis (Supplementary Figure S4). Again, weighting was effective in removing the association of prior non-RSV hospitalisation and comorbidities with nirsevimab immunisation (last columns of Supplementary Table S3).

Supplementary References

1. Hernán MA. How to estimate the effect of treatment duration on survival outcomes using observational data. *BMJ* 2018;360:k182. https://doi.org/10.1136/bmj.k182.
2. Hernán MA, Hernández-Díaz S, Robins JM. A structural approach to selection bias. *Epidemiology* 2004;15(5):615–625. https://doi.org/10.1097/01.ede.0000135174.63482.43.
3. Robins JM, Hernán MA, Brumback B. Marginal structural models and causal inference in epidemiology. *Epidemiology* 2000;11(5):550–560. https://doi.org/10.1097/00001648-200009000-00011.
4. Hernán MA, Brumback B, Robins JM. Marginal structural models to estimate the causal effect of zidovudine on the survival of HIV-positive men. *Epidemiology* 2000;11(5):561–570. https://doi.org/10.1097/00001648-200009000-00012.
5. Rose S, van der Laan MJ. A targeted maximum likelihood estimator for two-stage designs. *Int J Biostat* 2011;7(1):17. https://doi.org/10.2202/1557-4679.1217.
6. White H. A heteroskedasticity-consistent covariance matrix estimator and a direct test of heteroskedasticity. *Econometrica* 1980;48(4):817–838. https://doi.org/10.2307/1912934.
7. Rothman KJ, Greenland S, Lash TL. Case-control studies. In: Rothman KJ, Greenland S, Lash TL, editors. *Modern Epidemiology*, 3rd ed. Philadelphia: Wolters Kluwer/Lippincott Williams&Wilkins, 2008. https://solution.lww.com/book/isbn/9781451190052.

Supplementary Table S1. Start date of nirsevimab immunisation campaigns by autonomous region.

Autonomous region	Start of immunisation campaign
Andalusia†	September 25, 2023
Aragon*	October 2, 2023
Asturias	October 2, 2023
Balearic Islands*	November 27, 2023
Basque Country	November 13, 2023 (ended on February 29, 2024)
Canary Islands*	October 5, 2023
Cantabria	October 1, 2023
Castile and León	October 1, 2023
Castilla–La Mancha	October 2, 2023
Catalonia*	October 2, 2023
Ceuta	October 6, 2023
Extremadura*	October 30, 2023
Galicia	September 25, 2023
La Rioja	October 1, 2023
Madrid	October 1, 2023
Melilla	October 16, 2023 for catch-up immunisation<br>October 23, 2023 for at-birth immunisation
Murcia	September 25, 2023
Navarre	October 1, 2023 (ended on January 31, 2024)
Valencia	October 1, 2023

* Most autonomous regions participated with the whole public hospital network (the main healthcare provider in Spain), while Aragon, Balearic Islands, Catalonia, and Extremadura participated only with some public hospitals with better data accessibility and Canary Islands included only the three most populated islands.

† Most autonomous regions recruited all cases fulfilling the case definition, except Andalusia, which selected cases consecutively until reaching the final sample size.

Supplementary Table S2. Distribution of cases hospitalised for respiratory syncytial virus infection by autonomous region in the catch-up and at-birth immunisation studies.*

Autonomous region	Catch-up immunisation	At-birth immunisation
No. of cases	406 (100)	546 (100)
Andalusia	45 (11.1)	99 (18.1)
Aragon	7 (1.7)	12 (2.2)
Asturias	9 (2.2)	4 (0.7)
Balearic Islands†	22	1 (0.2)
Basque Country†	117	9 (1.6)
Canary Islands	34 (8.4)	21 (3.8)
Cantabria	0 (0.0)	4 (0.7)
Castile and León	19 (4.7)	31 (5.7)
Castilla–La Mancha	32 (7.9)	37 (6.8)
Catalonia	40 (9.9)	48 (8.8)
Ceuta	2 (0.5)	2 (0.4)
Extremadura†	9	5 (0.9)
Galicia	33 (8.1)	29 (5.3)
La Rioja	4 (1.0)	6 (1.1)
Madrid	75 (18.5)	80 (14.7)
Melilla†	1	5 (0.9)
Murcia	21 (5.2)	31 (5.7)
Navarre‡	15	14 (2.6)
Valencia	85 (20.9)	108 (19.8)

* Data are number (percentage).
† The regions of Balearic Islands, Basque Country, Extremadura, and Melilla were excluded from the catch-up study because their immunisation campaigns substantially overlapped with the respiratory syncytial virus epidemic.
‡ The region of Navarre was excluded from the catch-up study because catch-up immunisation was only implemented for children at high risk for respiratory syncytial virus infection.

Supplementary Table S3. Odds ratios for remaining unimmunised with nirsevimab by baseline and time-varying characteristics among previously unimmunised controls in the catch-up immunisation study.

Characteristic	Odds ratio* (95% CI)	P value	Weighted odds ratio† (95% CI)	P value
Sex				
Female	1.00 (reference)		1.00 (reference)	
Male	0.94 (0.84 to 1.05)	0.26	0.94 (0.84 to 1.06)	0.30
Gestational age (weeks)				
≥37	1.00 (reference)		1.00 (reference)	
<37	1.01 (0.79 to 1.28)	0.96	1.02 (0.80 to 1.30)	0.88
Birthweight (g)				
≥2500	1.00 (reference)		1.00 (reference)	
<2500	0.96 (0.75 to 1.23)	0.76	0.94 (0.74 to 1.20)	0.62
Multiple pregnancy				
No	1.00 (reference)		1.00 (reference)	
Yes	0.67 (0.40 to 1.12)	0.13	0.68 (0.40 to 1.15)	0.15
Previous non-RSV hospitalisation‡				
0–29 days of follow-up				
No	1.00 (reference)		1.00 (reference)	
Yes	0.90 (0.73 to 1.11)	0.32	1.01 (0.81 to 1.25)	0.96
≥30 days of follow-up				
No	1.00 (reference)		1.00 (reference)	
Yes	1.57 (0.76 to 3.26)	0.22	0.98 (0.61 to 1.59)	0.95
Previous comorbidities				
No	1.00 (reference)		1.00 (reference)	
Yes	1.09 (0.67 to 1.79)	0.72	1.02 (0.63 to 1.65)	0.94

* Odds ratios for remaining unimmunised with nirsevimab (95% confidence intervals) were obtained from a pooled logistic model among previously unimmunised controls, treating each child-day as an observation, allowing for different intercepts at 0–6, 7–14, 15–29, 30–59, 60–89, and ≥90 days, and adjusting for matching factors (random terms for province and natural cubic splines of birthdate with knots at the 5th, 27.5th, 50th, 72.5th, and 95th percentiles) and all categorical risk factors listed in the table. Conservative 95% confidence intervals and P values were calculated using robust standard errors.

† Weighted odds ratios (95% confidence intervals) were obtained from the same pooled logistic model with inverse probability of immunisation weights. Weights were calculated as the probability of having the observed immunisation history up to the current day given matching factors and baseline risk factors (sex, gestational age, birthweight, and multiple pregnancy), divided by the same probability further conditional on time-varying risk factors at previous day (previous non-RSV hospitalisation and previous comorbidities).

‡ Odds ratios for previous non-RSV hospitalisation varied with follow-up time and were modeled by including interaction terms between this risk factor and indicators of follow-up intervals (0–29 and ≥30 days).

Supplementary Table S4. Odds ratios for remaining unimmunised with nirsevimab by baseline and time-varying characteristics among previously unimmunised controls in the at-birth immunisation study.

Characteristic	Odds ratio* (95% CI)	P value	Weighted odds ratio† (95% CI)	P value
Sex				
Female	1.00 (reference)		1.00 (reference)	
Male	0.95 (0.85 to 1.07)	0.40	0.93 (0.83 to 1.04)	0.21
Gestational age (weeks)				
≥37	1.00 (reference)		1.00 (reference)	
<37	1.50 (1.12 to 1.99)	0.002	1.73 (1.27 to 2.34)	<0.001
Birthweight (g)				
≥2500	1.00 (reference)		1.00 (reference)	
<2500	1.83 (1.37 to 2.44)	<0.001	1.54 (1.16 to 2.06)	<0.001
Multiple pregnancy				
No	1.00 (reference)		1.00 (reference)	
Yes	1.09 (0.73 to 1.64)	0.64	1.18 (0.75 to 1.86)	0.42
Previous non-RSV hospitalisation‡				
0–2 days of follow-up				
No	1.00 (reference)		1.00 (reference)	
Yes	3.05 (2.16 to 4.31)	<0.001	1.03 (0.77 to 1.38)	0.84
3–6 days of follow-up				
No	1.00 (reference)		1.00 (reference)	
Yes	0.85 (0.59 to 1.22)	0.36	1.01 (0.59 to 1.75)	0.95
≥7 days of follow-up				
No	1.00 (reference)		1.00 (reference)	
Yes	0.28 (0.19 to 0.42)	<0.001	1.51 (0.64 to 3.56)	0.14
Previous comorbidities				
No	1.00 (reference)		1.00 (reference)	
Yes	5.41 (1.89 to 15.5)	0.002	1.39 (0.33 to 5.85)	0.56

* Odds ratios for remaining unimmunised with nirsevimab (95% confidence intervals) were obtained from a pooled logistic model among previously unimmunised controls, treating each child-day as an observation, allowing for different intercepts at 0, 1, 2, 3–6, 7–13, and ≥14 days, and adjusting for matching factors (random terms for province and natural cubic splines of birthdate with knots at the 5th, 27.5th, 50th, 72.5th, and 95th percentiles) and all categorical risk factors listed in the table. Conservative 95% confidence intervals and P values were calculated using robust standard errors.

† Weighted odds ratios (95% confidence intervals) were obtained from the same pooled logistic model with inverse probability of immunisation weights. Weights were calculated as the probability of having the observed immunisation history up to the current day given matching factors and baseline risk factors (sex, gestational age, birthweight, and multiple pregnancy), divided by the same probability further conditional on time-varying risk factors at previous day (previous non-RSV hospitalisation and previous comorbidities).

‡ Odds ratios for previous non-RSV hospitalisation varied with follow-up time and were modeled by including interaction terms between this risk factor and indicators of follow-up intervals (0–2, 3–6, and ≥7 days).

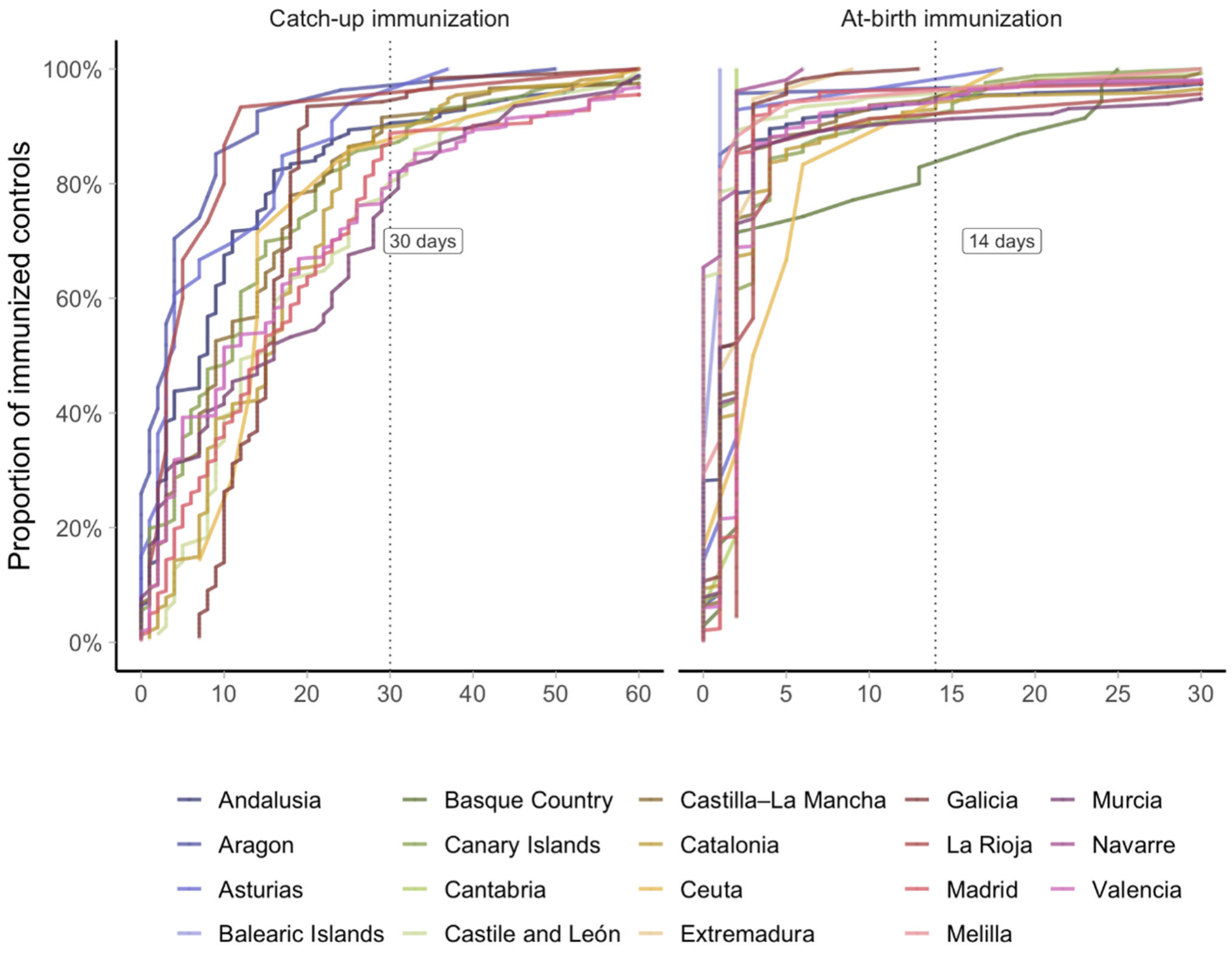

Supplementary Figure S1. Proportion of controls immunised since the onset of nirsevimab immunisation campaigns in the catch-up immunisation study and since birth in the at-birth immunisation study by autonomous region.

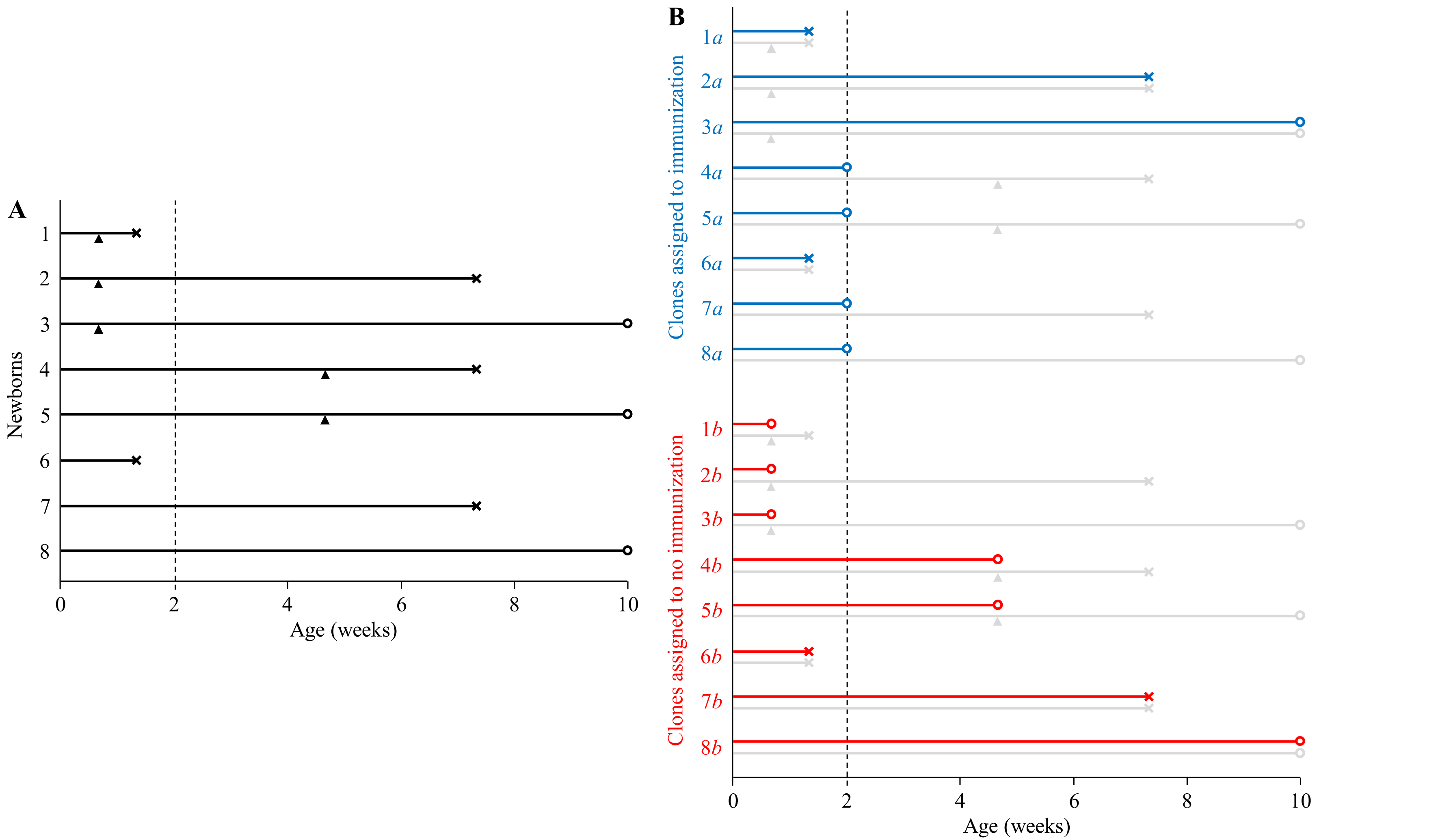

Supplementary Figure S2. Follow-up of newborns in the underlying population (A) and their clones assigned to either immunisation or no immunisation for the per-protocol analysis (B). Symbols represent nirsevimab immunisation (▲), hospitalisation for respiratory syncytial virus infection (×), and censoring (○).

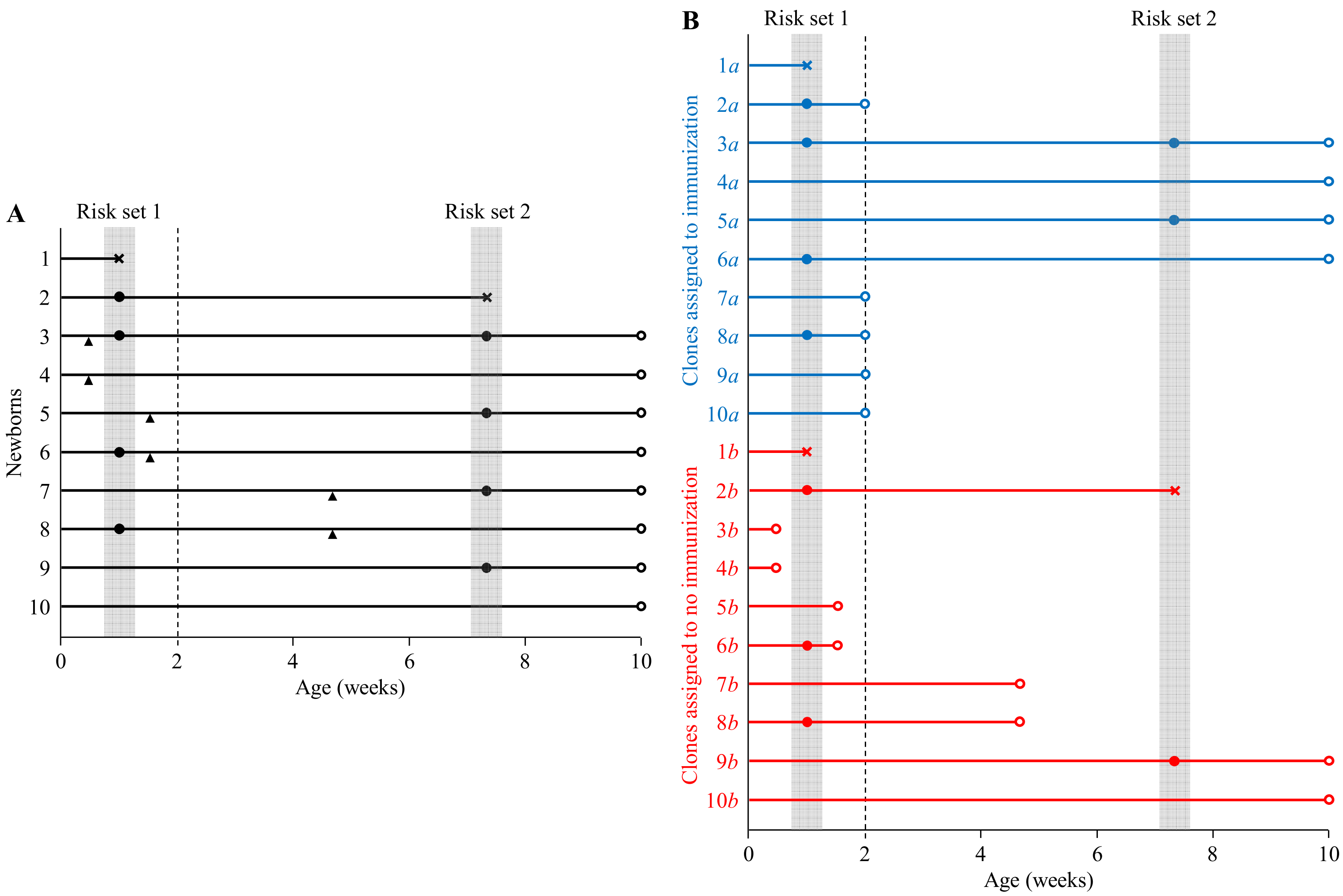

Supplementary Figure S3. Risk sets of newborns in the underlying population (A) and their clones assigned to either immunisation or no immunisation for the per-protocol analysis (B). Symbols represent nirsevimab immunisation (▲), hospitalisation for respiratory syncytial virus infection (×), control selection (●), and censoring (○).

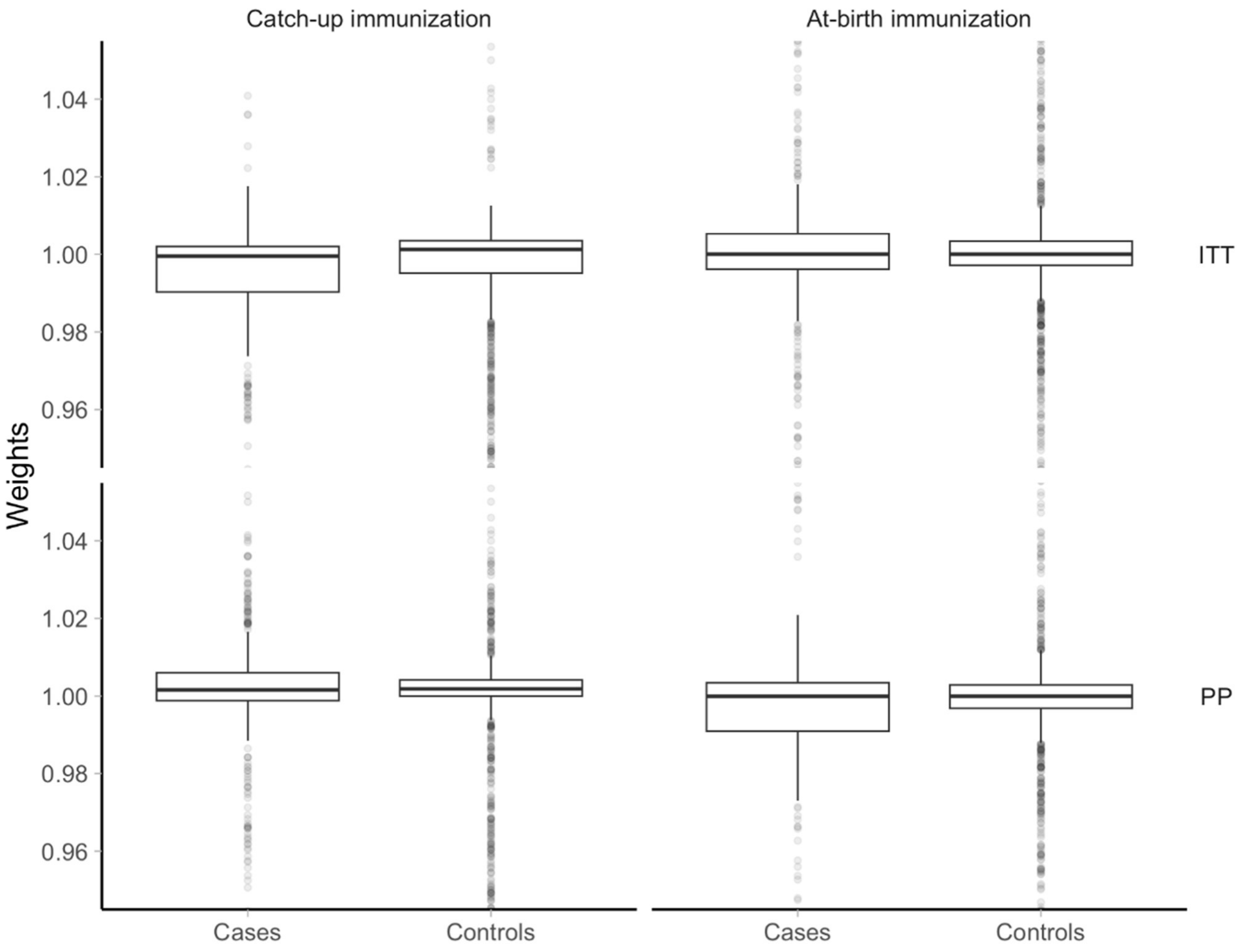

Supplementary Figure S4. Distribution of inverse probability of censoring weights among cases and controls for intention-to-treat (ITT) and per-protocol (PP) analyses in the catch-up and at-birth immunisation studies.
